# Supplementary material for: Transcriptome Analysis Unveiled the Intricate Interplay between Sugar Metabolism and Lipid Biosynthesis in Symplocos paniculate Fruit
Source: Plants (Basel). 2023 Jul 20;12(14):2703. doi: 10.3390/plants12142703 (PMC10385272; doi:10.3390/plants12142703)
Supplement: Supplementary file 1 [file plants-12-02703-s001.zip › plants-2477912-supplementary.pdf]

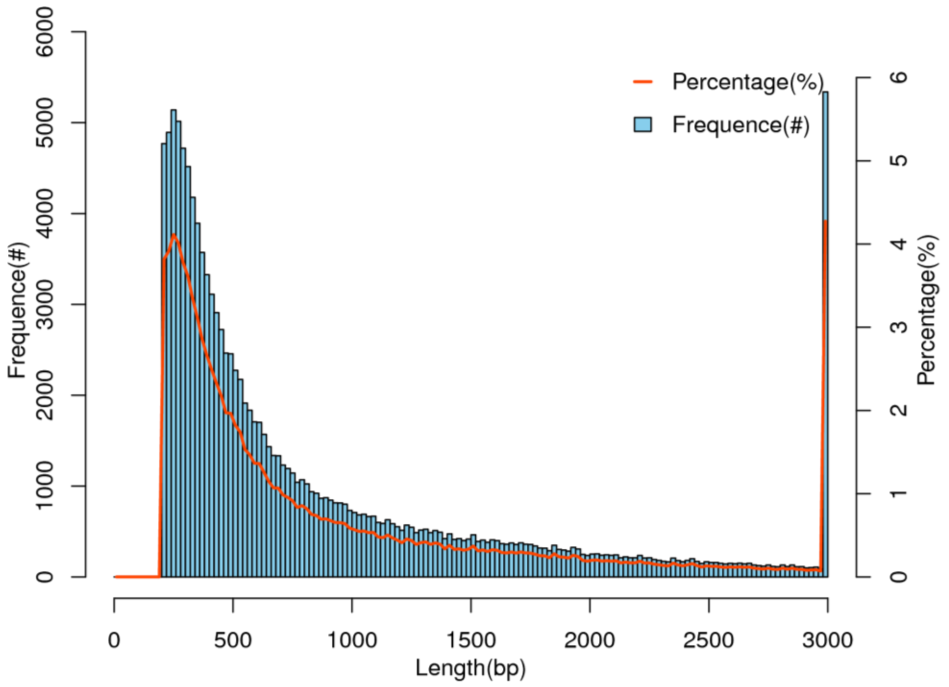

Figure S1. Frequency of *Symplocos paniculata* unigenes.

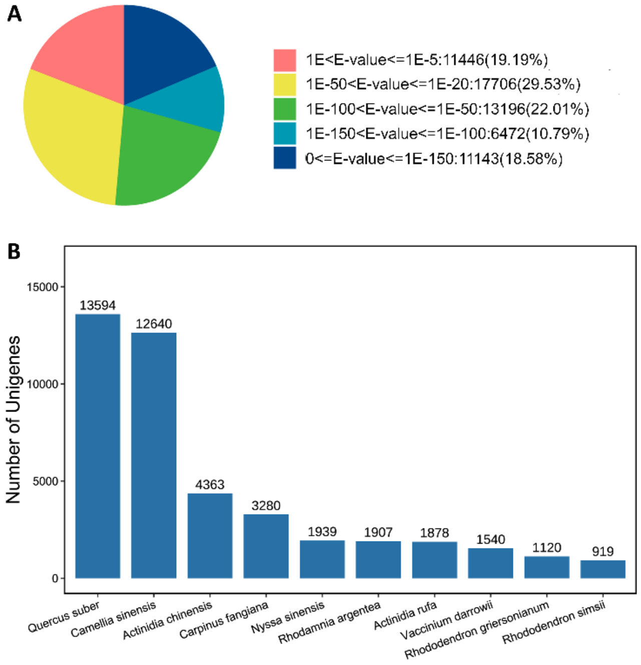

Figure S2. Similarity analysis between *Symplocos paniculata* unigenes and NR database. E-value (<1E-5) distribution of top BLAST hits for each *S. paniculata* unigene, B. Top-hit species distribution for BLAST matches for *S. paniculata* unigenes

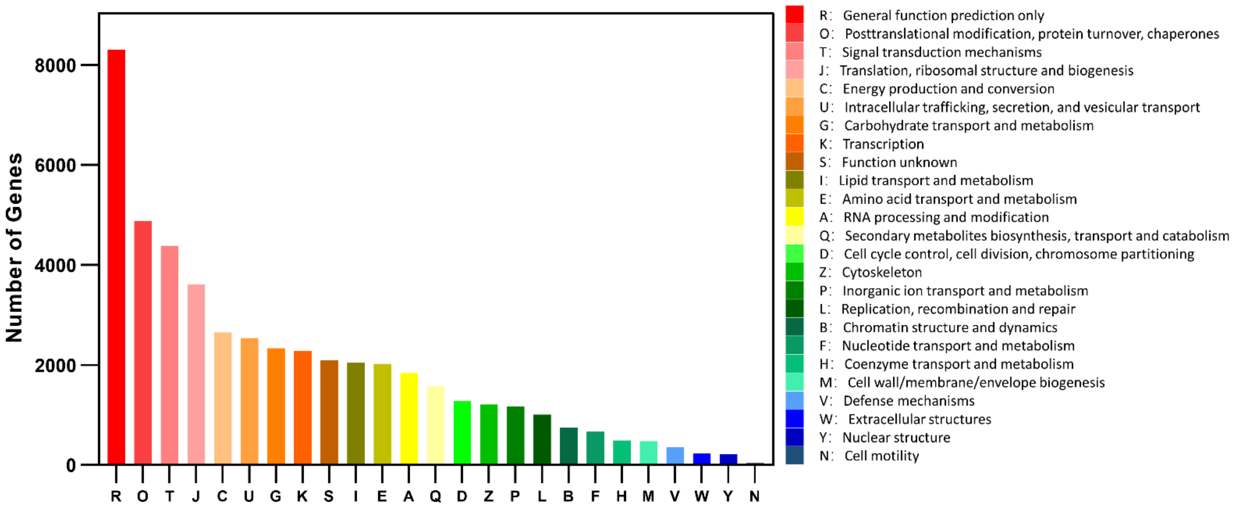

Figure S3. COG classifications of *Symplocos paniculata* unigenes.

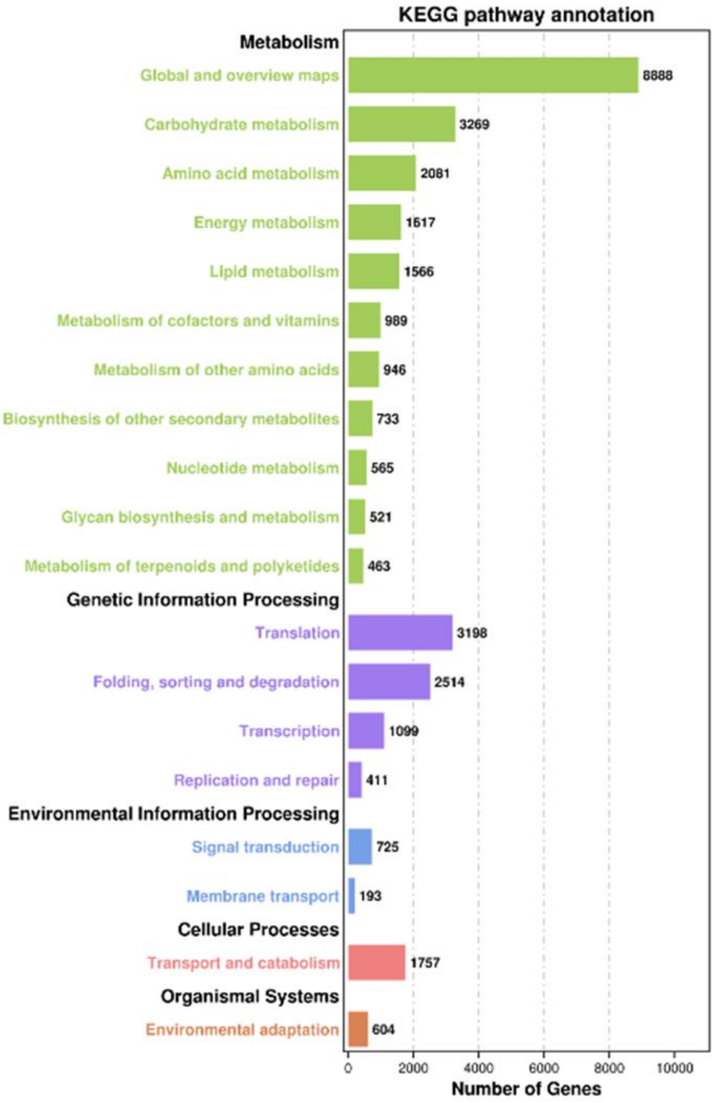

Figure S4. KEGG function annotation classification in *Symplocos paniculata* unigenes.

Table S1 Gene primer sequence

Gene ID	Gene name	Forward (5'-3')	Reverse (5'-3')
Unigene0054085	PGK3	GTGAGGCCTTGGATACCACC	ATCGTTGTCACCCCCTTGTC
Unigene0010619	PKP1	TTGCCTCTCAGCTGCTTGAA	GCATCTGCTCGTTGCCTTAC
Unigene0088893	PDH-E1	GATCACGTTCACGCGTTGAG	GTCACATGATCGCAATCGGC
Unigene0135681	MDH	AGGTAGCTGGGTACATGGGT	ACAACTGCATGGGGGCAATA
Unigene0081204	FATB	ACCATGTGAAGAGTGCAGGG	ACTTGCACAACATCACCCCA
Unigene0091410	DGAT3	GCAAGTGCAGAGATGGTCCT	TGGCCACAATTAAGCCCACA
Unigene0087829	MAT	TAAGGGGTGAAGCCATGCAG	GAGACAGCCGGTTCCATGAA
Unigene0117377	LPAAT	GCGCAATCCTCTCCTACCTC	CCAAGCATCCCCACTCATGT
	ALB	ACCCTGCCAGTGGATTTATG	CCTTATGCCTACGGTTTTGC
	ETIF3H	GACACCTTGGGAGGACTTTG	GTGGATAATAATACTGTTGGATGG

Table S2 The raw sequencing data of *Symplocos paniculata*

Sample	Raw Data (bp)	Clean Data (bp)	AF_Q30(%)	AF_GC(%)	Unique_Mapped(%)	Total_Mapped(%)
BT_PULP_A-1	7552712100	7444281036	6786109177 (91.16%)	3396754539 (45.63%)	39078568 (78.08%)	40919187 (81.76%)
BT_PULP_A-2	6996553200	6888920888	6322770781 (91.78%)	3179733237 (46.16%)	36071253 (77.89%)	37934997 (81.91%)
BT_PULP_A-3	7846000500	7734968181	7105051486 (91.86%)	3541818256 (45.79%)	40578818 (78.04%)	42586075 (81.90%)
BT_PULP_B-1	8463280200	8334993351	7623592272 (91.46%)	3965665819 (47.58%)	43942123 (78.34%)	46898367 (83.61%)
BT_PULP_B-2	6933796200	6853760906	6313433773 (92.12%)	3311715318 (48.32%)	36327655 (79.03%)	38775059 (84.35%)
BT_PULP_B-3	6891328200	6777663894	6186381459 (91.28%)	3212168408 (47.39%)	35743677 (78.32%)	37948868 (83.15%)
BT_PULP_C-1	7939704000	7843148264	7173750692 (91.47%)	3890996368 (49.61%)	42013595 (79.85%)	45759590 (86.96%)
BT_PULP_C-2	7857146100	7787124806	7141686408 (91.71%)	3861885709 (49.59%)	41782417 (80.23%)	45214245 (86.82%)
BT_PULP_C-3	8175102000	8073953465	7300636094 (90.42%)	3965626651 (49.12%)	43138229 (79.71%)	46597972 (86.10%)
BT_SEED_A-1	9347962500	9207622302	8623752806 (93.66%)	4074662200 (44.25%)	47715558 (76.95%)	49679022 (80.12%)
BT_SEED_A-2	7648063200	7532362362	6958785312 (92.39%)	3367787694 (44.71%)	39329090 (77.63%)	40903492 (80.74%)
BT_SEED_A-3	7256855400	7143966581	6538966179 (91.53%)	3151720452 (44.12%)	37113678 (77.16%)	38563820 (80.18%)
BT_SEED_B-1	7948328400	7838469865	7183065136 (91.64%)	3479583319 (44.39%)	43223769 (82.08%)	44602770 (84.69%)
BT_SEED_B-2	7054966800	6988131839	6397629384 (91.55%)	3235235355 (46.30%)	38836570 (83.09%)	39901024 (85.37%)
BT_SEED_B-3	7667886000	7530154469	6913425772 (91.81%)	3465628964 (46.02%)	41196751 (81.05%)	42528746 (83.68%)
BT_SEED_C-1	7858345500	7777490141	7165562303 (92.13%)	3496542481 (44.96%)	41083550 (78.91%)	42909858 (82.42%)
BT_SEED_C-2	6160595700	6083255088	5555047306 (91.32%)	2855320903 (46.94%)	32021486 (78.47%)	33320657 (81.66%)
BT_SEED_C-3	8108864700	8018316208	7307377511 (91.13%)	3650140877 (45.52%)	42269633 (78.75%)	43969627 (81.91%)

Table S3 Summary of the sequencing data of *Symplocos paniculata*

Genes Num	GC percentage	N50 number	N50 length	Max length	Min length	Average length	Total assembled bases
124923	41.4909	22544	1480	18668	201	915	114404745

Table S4 Analysis of the differences in metabolic pathways between pulp and seed carbohydrates in *Symplocos paniculata* fruit.

KEGG-pathway	Pulp vs Seed		
	90 DAF	120 DAF	140 DAF
Glycolysis / Gluconeogenesis	74	124	230
Pyruvate metabolism	59	108	209
Starch and sucrose metabolism	72	90	173
Glyoxylate and dicarboxylate metabolism	47	84	172
Amino sugar and nucleotide Carbohydrate metabolism	52	62	161
Citrate cycle（TCA cycle）	11	38	133
Pentose and glucuronate interconversions	37	45	124
Pentose phosphate pathway	27	46	104
Fructose and mannose metabolism	31	50	103
Ascorbate and aldarate metabolism	40	59	99
Propanoate metabolism	6	23	57
Galactose metabolism	26	34	88
Inositol phosphate metabolism	16	26	73
Butanoate metabolism	9	20	50
C5-Branched dibasic acid metabolism	1	5	12
Fatty acid metabolism	23	50	158
Fatty acid degradation	32	57	110
Glycerolipid metabolism	36	59	130
Glycerophospholipid metabolism	19	38	122
Fatty acid biosynthesis	13	33	70
Biosynthesis of unsaturated fatty acids	10	11	59
Sphingolipid metabolism	10	15	69
alpha-Linolenic acid metabolism	19	18	52
Steroid biosynthesis	13	18	58
Ether lipid metabolism	7	14	34
Fatty acid elongation	16	12	40
Arachidonic acid metabolism	3	12	25
Cutin, suberine and wax biosynthesis	11	18	22
Linoleic acid metabolism	6	8	9

Table S5 Correlations between FPKM with ΔΔCt using qRT-PCR of key enzymes

Unigene ID	Abbreviation	Enzymes name	Correlation coefficient
Unigene0054085	PGK3	Phosphoglycerate kinase	0.9758
Unigene0010619	PKP1	Pyruvate kinase	0.8589
Unigene0088893	PDH-E1	Pyruvate dehydrogenase	0.9213
Unigene0135681	MDH	Malate dehydrogenase	0.7996
Unigene0081204	FATB	Acyl ACP thiolipase B	0.8561
Unigene0091410	DGAT3	Diacylglycerol acyltransferase	0.9211
Unigene0087829	MAT	ACP malonyltransferase	0.9633
Unigene0117377	LPAAT	Lysophosphatidyltransferase	0.8314
